# Relationship between time of emergency department admission and adherence to the Surviving Sepsis Campaign bundle in patients with septic shock

**DOI:** 10.1186/s13054-022-03899-0

**Published:** 2022-02-11

**Authors:** Je Sung You, Yoo Seok Park, Sung Phil Chung, Hye Sun Lee, Soyoung Jeon, Won Young Kim, Tae Gun Shin, You Hwan Jo, Gu Hyun Kang, Sung Hyuk Choi, Gil Joon Suh, Byuk Sung Ko, Kap Su Han, Jong Hwan Shin, Taeyoung Kong, Korean Shock Society (KoSS) Investigators, Won Young Kim, Seung Mok Ryoo, Tae Gun Shin, Sung Yeon Hwang, You Hwan Jo, Sung Phil Chung, Yoon Jung Hwang, Jin Ho Beom, Yoo Seok Park, Gu Hyun Kang, Sung-Hyuk Choi, Young-Hoon Yoon, Gil Joon Suh, Tae Ho Lim, Byuk Sung Ko, Kap Su Han, Jong Hwan Shin, Hui Jai Lee, Kyoung Min You

**Affiliations:** 1 Department of Emergency Medicine, Yonsei University College of Medicine, 211, Eonju-ro, Gangnam-gu, Seoul, 06273 Republic of Korea; 2 Department of Research Affairs, Biostatistics Collaboration Unit, Yonsei University College of Medicine, Seoul, 06273 Republic of Korea; 3 Department of Emergency Medicine, University of Ulsan College of Medicine, Asan Medical Center, Seoul, Republic of Korea; 4 Department of Emergency Medicine, Samsung Medical Center, Sungkyunkwan University School of Medicine, Seoul, Republic of Korea; 5 Department of Emergency Medicine, Seoul National University Bundang Hospital, Seongnam, Republic of Korea; 6 Department of Emergency Medicine, Hallym University College of Medicine, Seoul, Republic of Korea; 7 Department of Emergency Medicine, Guro Hospital, Korea University Medical Center, Seoul, Republic of Korea; 8 Department of Emergency Medicine, Seoul National University College of Medicine, Seoul, Republic of Korea; 9 Department of Emergency Medicine, College of Medicine, Hanyang University, Seoul, Republic of Korea; 10 Department of Emergency Medicine, Korea University Anam Hospital, Seoul, Republic of Korea; 11 Department of Emergency Medicine, Seoul National University Boramae Medical Center, Seoul, Republic of Korea; 12 University of Ulsan College of Medicine, Asan Medical Center, Seoul, Republic of Korea; 13 Samsung Medical Center, Sungkyunkwan University School of Medicine, Seoul, Republic of Korea; 14 Seoul National University Bundang Hospital, Seongnam, Republic of Korea; 15 Yonsei University College of Medicine, Seoul, Republic of Korea; 16 University College of Medicine, Seoul, Republic of Korea; 17 Guro Hospital, Korea University Medical Center, Seoul, Republic of Korea; 18 Seoul National University Hospital, Seoul, Republic of Korea; 19 College of Medicine, Hanyang University, Seoul, Republic of Korea; 20 Korea University Anam Hospital, Seoul, Republic of Korea; 21 Seoul National University Boramae Medical Center, Seoul, Republic of Korea

**Keywords:** Sepsis, Septic shock, Off-hour effect, Surviving Sepsis Campaign

## Abstract

**Background:**

Nighttime hospital admission is often associated with increased mortality risk in various diseases. This study investigated compliance rates with the Surviving Sepsis Campaign (SSC) 3-h bundle for daytime and nighttime emergency department (ED) admissions and the clinical impact of compliance on mortality in patients with septic shock.

**Methods:**

We conducted an observational study using data from a prospective, multicenter registry for septic shock provided by the Korean Shock Society from 11 institutions from November 2015 to December 2017. The outcome was the compliance rate with the SSC 3-h bundle according to the time of arrival in the ED.

**Results:**

A total of 2049 patients were enrolled. Compared with daytime admission, nighttime admission was associated with higher compliance with the administration of antibiotics within 3 h (adjusted odds ratio (adjOR), 1.326; 95% confidence interval (95% CI), 1.088–1.617, *p* = 0.005) and with the complete SSC bundle (adjOR, 1.368; 95% CI, 1.115–1.678; *p* = 0.003), likely to result from the increased volume of all patients and sepsis patients admitted during daytime hours. The hazard ratios of the completion of SSC bundle for 28-day mortality and in-hospital mortality were 0.750 (95% CI 0.590–0.952, *p* = 0.018) and 0.714 (95% CI 0.564–0.904, *p* = 0.005), respectively.

**Conclusion:**

Septic shock patients admitted to the ED during the daytime exhibited lower sepsis bundle compliance than those admitted at night. Both the higher number of admitted patients and the higher patients to medical staff ratio during daytime may be factors that are responsible for lowering the compliance.

**Supplementary Information:**

The online version contains supplementary material available at 10.1186/s13054-022-03899-0.

## Background

Each year, approximately 850,000 adult patients are admitted to the emergency department (ED) in the USA for sepsis or septic shock [1]. The Surviving Sepsis Campaign (SSC), which aims to improve clinical outcomes in patients being treated for sepsis, has established and endorsed international clinical practice guidelines for the management of sepsis or septic shock [2, 3]. These guidelines consist of a bundle that combines treatments for the various components of sepsis, such as rapid fluid resuscitation, timely and appropriate administration of antibiotics following blood sample collection for culture, the use of vasopressors to maintain arterial pressure, and quantification of lactate concentrations [4]. For sepsis patients, compliance with SSC bundles has remained the cornerstone for improving quality and clinical outcomes since the publication of the first SSC guidelines [3].

Owing to certain uncontrollable variables, the off-hour or nighttime effect is usually defined as an increased risk of mortality during off-hour admissions for the treatment of various diseases or conditions [5–7]. For example, many studies have demonstrated the adverse effects of off-hours admissions on diagnosis, treatment, and clinical outcomes in several diseases requiring time-sensitive interventions, such as polytrauma, myocardial infarction, and stroke [8]. Compared with daytime hours, medical services in hospitals are commonly reduced at night due to a shortage of staff, lack of experienced clinicians, diminished access to hospital services and resources, and inadequate subspecialty care [8–10]. However, for patients visiting the ED with sepsis, crowding may also affect adherence to treatment bundles, some components of which are time-sensitive in nature [11]. In a multicenter study of sepsis patients in EDs, ED crowding was associated with a delay in initial patient assessments and antibiotic administration [11]. In addition, the diurnal variation in ED crowding was observed, with the lowest occupancy being from midnight to early morning hours [11, 12].

Considering these opposite factors, whether nighttime admission can adversely affect timely SSC bundle management is debatable. Few studies have evaluated the association between adequacy of treatment and the time of ED visits in patients with sepsis, and conflicting results have been reported [13]. Therefore, this large, multicenter study was conducted to investigate the rate of compliance with the SSC 3-h bundle for nighttime and daytime ED admissions and to investigate the clinical impacts of compliance on mortality in patients with septic shock.

## Methods

### Study design and population

We conducted an observational study using a prospective, multicenter registry of septic shock data provided by the Korean Shock Society (KoSS) related to patients treated from November 2015 to December 2017. The KoSS web-based septic shock registry has been prospectively collecting predetermined data pertaining to patients with septic shock who visited the EDs of 11 teaching hospitals throughout South Korea since October 2015 [14–16]. All data were collected using standardized web-based electronic case report forms by research coordinators located in each individual institution; this consisted of standard definitions of approximately 200 variables, including clinical characteristics, laboratory and time-related data, therapeutic interventions, and the outcomes of patients treated for septic shock [16]. The study design was reviewed and approved by the institutional review boards of the individual participating institutions prior to the initiation of data collection. Patients from the septic shock registry who were aged > 18 years and who met the inclusion criteria were enrolled. As the implementation of the KoSS registry began prior to the publication of the Sepsis-3 criteria, the inclusion criteria were based on evidence of refractory hypotension or hyperlactatemia in patients with suspected or confirmed infection. In the present study, we defined hypotension as systolic blood pressure (SBP) < 90 mmHg, a mean arterial pressure < 65 mmHg, or an SBP decrease > 40 mmHg. Refractory hypotension was defined as persistent hypotension based on the same values following an adequate intravenous fluid challenge (20–30 mL/kg or at least 1 L of a crystalloid solution administered over 30 min) or as the need for vasopressors following fluid resuscitation [14–16]. Hypoperfusion was defined as a serum lactate concentration of ≥ 4 mmol/L [14–16]; these levels were routinely assessed when the shock was suspected or after a fluid challenge was administered.

The following patients were not enrolled in the KoSS registry: patients who did not meet the inclusion criteria within 6 h following ED admission; patients who were transferred from other hospitals without meeting the inclusion criteria upon ED admission or who were transferred from the ED to other hospitals; and patients who signed a “do not attempt resuscitation” order. In 2013 when the KoSS was organized, the SSC 6-h bundle was implemented in South Korea as the standard protocol for sepsis management in EDs of almost all institutions. To minimize disparities in therapeutic effects resulting from the implementation of different protocols among hospitals, we excluded patients who met the inclusion criteria 6 h after arriving in the ED, as well as patients who were not provided information about sepsis bundle management or survival outcomes.

### Data collection

We retrieved all the demographic and clinical data of all subjects in this study, including age, sex, past medical history, initial vital signs, laboratory values upon ED admission, Sequential Organ Failure Assessment (SOFA) score, Acute Physiologic Assessment and Chronic Health Evaluation (APACHE) II score, therapeutic interventions, and clinical outcomes from the KoSS registry.

Compliance with individual components of the SSC bundle was also recorded in this registry, which included the following procedures: quantification of serum lactate concentration, lactate clearance and duration between first and second measurements of lactate, fluid resuscitation, administration of vasopressors to maintain mean arterial pressure ≥ 65 mmHg, collection of blood samples or other specimens for appropriate culturing, and antibiotic administration. The daytime and nighttime were determined based on average duty shift, which are the times with the greatest change in the number of medical personnel in ED of 11 participated institutions. Enrolled patients were classified into two groups based on their time of arrival at the ED, either during the day (09:00 to 18:59) or at night (19:00 to 08:59).

In addition, we collected information about patient volume at the time of ED admissions of 11 participating institutions from the Korea National Emergency Department Information System (NEDIS) database [17]. The NEDIS is a nationwide government-run system that collects the clinical and administrative data from all EDs designated by the Ministry of Health and Welfare of Korea. The NEDIS contains patient data including sex, age, type of insurance, means of transportation, level of consciousness at presentation, emergency operative procedures, time variables (visit, discharge, and admission), critical care requirement, disposition status after the ED encounter, hospital stay after admission, and final outcomes (information regarding discharge, transfer, and death) [18]. To identify the available information about infrastructure per duty-time for 11 participating institutions, we identified the volume of ED visiting patients and patients/medical staff ratio, according to time zone or each hospital.

### Study outcomes

The primary outcome was defined as the completion of the SSC 3-h bundle, which comprises lactate measurements, blood draws for culturing prior to antibiotic administration, prompt administration of broad-spectrum antibiotics, and appropriate fluid challenge for patients with a mean arterial pressure < 65 mmHg and/or a serum lactate concentration of 4 mmol/L or greater [13]. Secondary outcomes were adherence to the individual components of the SSC 3-h bundle. The compliance rate with the SSC 3-h bundle was calculated based on the time of arrival in the ED. In addition, in-hospital and 28-day mortality were also analyzed according to compliance with the SSC 3-h bundle.

### Statistical analyses

Demographic and clinical data are presented as median values with interquartile ranges, means ± standard deviations (SDs), percentages, or frequencies, as appropriate. Continuous variables were compared using two-sample *t* tests or Mann–Whitney U tests for parametric and nonparametric variables, respectively. Categorical variables were compared using Chi-square or Fisher’s exact tests. Any missing data were not replaced. Univariable analyses were conducted to evaluate the relationships between clinical characteristics and adherence to individual components of the SSC bundle. To identify independent factors affecting compliance with individual components of the bundle, multivariable logistic regression analyses were conducted, integrating the major covariates identified from the univariate analyses (i.e., variables with a *p* < 0.05), prior knowledge and clinical plausibility. To identify independent factors affecting compliance with complete SSC bundle according to daytime and nighttime arrivals. We conducted stratified logistic regression (strata: hospital) using hospitals as stratification factors under the assumption that each hospital had different characteristics and underlying risks. This analysis also revealed that nighttime admission had an independent association with the improved performance in SSC bundle by these results. Depending on the severity of disease, we performed sensitivity analysis to identify differences in compliance to the SSC bundle by clinical severity. We divided the patients into subgroups as follows: SOFA score < 8 or SOFA score ≥ 8 and Lactate level < 4 or Lactate level ≥ 4. We identified the association between nighttime admission and compliance of SSC bundle performance. Using univariate and multivariable Cox proportional hazards regression analyses, the independent prognostic factors related to in-hospital and 28-day mortality rates were determined based on the compliance rates of the individual components of the SSC bundle. Kaplan–Meier survival curves and the log-rank test were used to identify significant relationships between the adherence to the SSC 3-h bundle, in-hospital mortality and 28-day mortality. The results are expressed as odds ratios (ORs) or hazard ratio (HR) and 95% confidential intervals (CIs). Statistical analyses were performed using SAS, version 9.2 (SAS Institute Inc., Cary, NC) and MedCalc Statistical Software version 16.4.3 (MedCalc Software bvba, Ostend, Belgium). *p* values < 0.05 were considered statistically significant.

## Results

### Participant characteristics

**Fig. 1:**
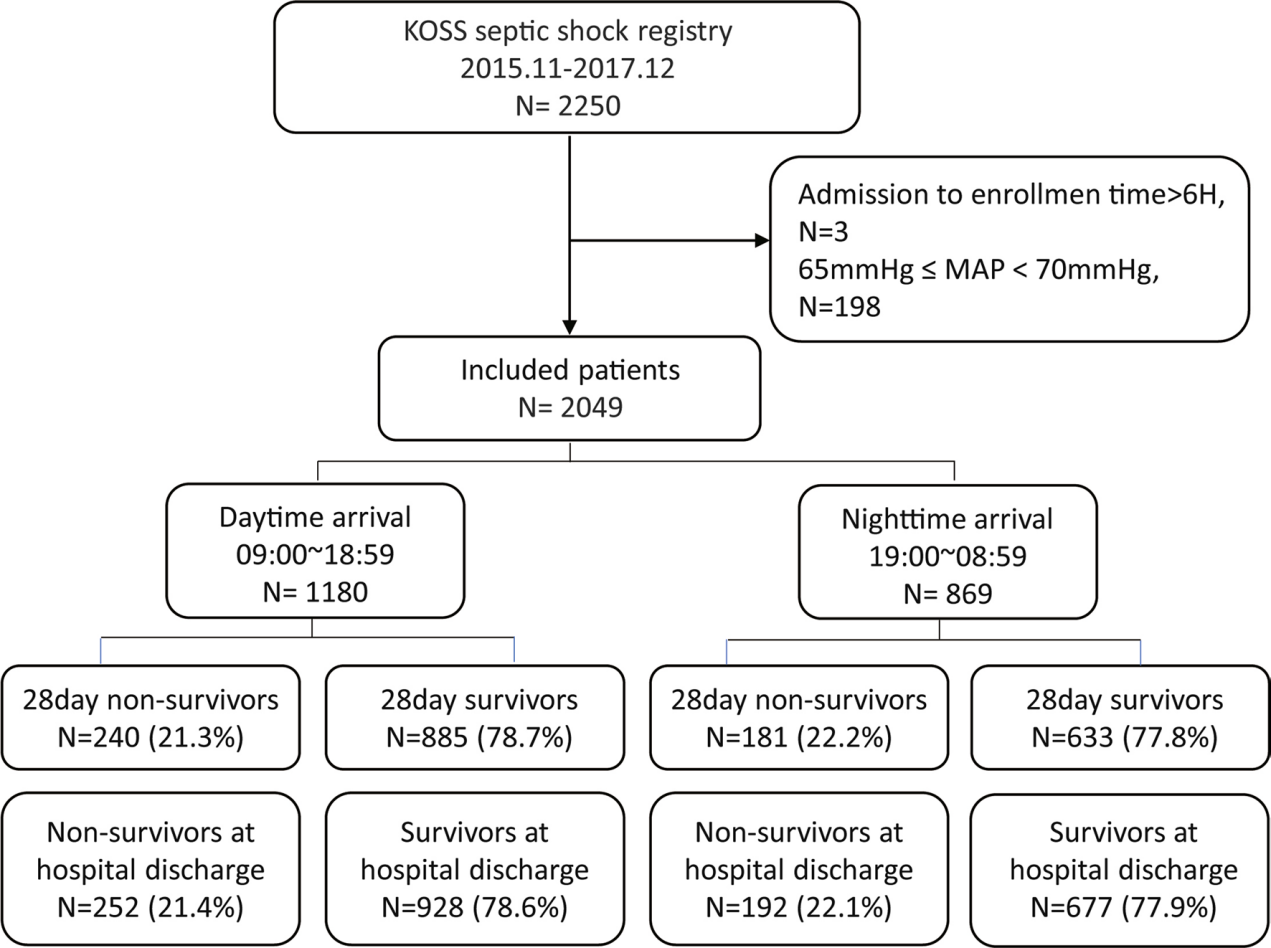
Flow diagram of patient inclusion and exclusion. *KoSS* Korean Shock Society, *MAP* mean arterial pressure

During the study period, data from 2347 patients were registered in the KoSS registry. A total of 298 patients were excluded from the analysis according to the predetermined criteria. After exclusion, a total of 2049 patients with septic shock were enrolled in this study. The enrollment and clinical outcome data for patients with septic shock are shown in the flow diagram in Fig. 1 (Fig. 1).

The eligible patients were stratified based on whether they visited the ED during the day (1180; 58%) or night (869; 42%). Table 1 shows the comparison of clinical characteristics of the patients with septic shock between those who arrived at the ED during the day or at night. There were no significant differences between the two groups in terms of age, sex, SOFA score, APACHEII score, intensive care unit (ICU) admission rate, or the 28-day or in-hospital mortality rates (Table 1). The standardized sepsis protocol (Code Sepsis) was applied in 9 institutions, and the protocol did not exist in two institutions (Additional file 1: Table S1). However, in the two institutions, timely administration of antibiotics was constantly monitored by quality indicators to ensure patients’ safety and meet guidance of Korea national insurance. There were higher adherence of timely antibiotic administration, lactate measurement, and blood cultures in institutions without standardized sepsis protocol. However, there were lower adherence of timely fluid and vasopressor administration. Regardless of the application of standardized sepsis protocol, there was no difference adherence of full SSC bundle (Additional file 1: Table S2).

**Table 1:** Comparison of demographic and clinical characteristics for daytime versus nighttime admissions of all patients admitted to the emergency department and those with septic shock

Variables	Total	Day	Night	*P*
	*N* = 2049 (100%)	*N* = 1180 (58%)	*N* = 869 (42%)	
Age (years)	67.9 ± 13.6	68.1 ± 13.4	67.7 ± 14.0	0.595
Male sex [*n* (%)]	1196 (58.4)	684 (58.0)	512 (58.9)	0.666
Severity score				
SOFA score (points)	6.07 ± 3.16	6.11 ± 3.15	6.02 ± 3.17	0.567
APACHE II score (points)	20.24 ± 9.11	20.31 ± 9.15	20.15 ± 9.07	0.703
Initial vital sign				
Systolic blood pressure (mmHg)	89.2 ± 23.9	88.9 ± 22.8	89.6 ± 25.4	0.567
Diastolic blood pressure (mmHg)	53.8 ± 16.2	53.5 ± 15.3	54.1 ± 17.3	0.411
Body temperature (°C)	37.7 ± 1.3	37.6 ± 1.3	37.7 ± 1.4	0.118
Past medical history [*n* (%)]				
Hypertension	846 (41.3)	490 (41.5)	356 (41.0)	0.799
Diabetes mellitus	627 (30.6)	348 (29.5)	279 (32.1)	0.204
Cardiovascular disease	275 (13.4)	166 (14.1)	109 (12.5)	0.317
Cerebrovascular disease	250 (12.2)	148 (12.5)	102 (11.7)	0.582
Chronic lung disease	164 (8.0)	103 (8.7)	61 (7.0)	0.159
Hematologic malignancy	134 (6.5)	78 (6.6)	56 (6.4)	0.881
Metastatic cancer	452 (22.1)	245 (20.8)	207 (23.8)	0.099
Chronic kidney disease	151 (7.4)	86 (7.3)	65 (7.5)	0.869
Chronic liver disease	242 (11.8)	128 (10.9)	114 (13.1)	0.115
Transplantation	39 (1.9)	22 (1.9)	17 (2.0)	0.881
AIDS	5 (0.2)	3 (0.3)	2 (0.2)	> 0.999
Source of infection [*n* (%)]				
GI tract	360 (17.6)	184 (15.6)	176 (20.3)	0.006*
Hepatobiliary or pancreas	239 (11.7)	137 (11.6)	102 (11.7)	0.929
Respiratory	53 (2.6)	31 (2.6)	22 (2.5)	0.893
Soft tissue/bone/joint	123 (6.0)	75 (6.4)	48 (5.5)	0.433
Urinary	1241 (60.6)	684 (58.0)	557 (64.1)	0.005*
Mixed	105 (5.1)	65 (5.5)	40 (4.6)	0.358
Unknown	386 (18.8)	221 (18.7)	165 (19.0)	0.882
Laboratory data				
White blood cell count (10^3^/μL)	13.2 ± 16.7	13.8 ± 18.6	12.4 ± 13.7	0.047*
C-reactive protein (mg/L)	14.42 ± 12.68	15.21 ± 13.51	13.35 ± 11.37	< 0.001*
Lactate (mmol/L)	4.37 ± 3.31	4.22 ± 3.21	4.58 ± 3.42	0.017*
Lactate clearance (%)	10.0 ± 58.8	9.6 ± 62.3	10.7 ± 54.1	0.743
Duration of lactate measurement (H)	2.6 ± 1.5	2.7 ± 1.5	2.4 ± 1.5	0.010*
Volume of patients				
Patients/doctor ratio	1.19 ± 0.44	1.25 ± 0.33	1.10 ± 0.54	< 0.001*
Patients/nurse ratio	0.86 ± 0.32	0.95 ± 0.28	0.73 ± 0.33	< 0.001*
Number of patients per hour (n)	60,838 ± 21,159	74,929 ± 6495	50,773 ± 22,384	0.002*
Ratio of patients per hour (%)	4.67 ± 1.26	5.24 ± 0.59	3.89 ± 1.49	< 0.001*
Clinical outcomes [*n* (%)]				
28-day mortality	421 (21.71)	240 (21.33)	181 (22.24)	0.634
In-hospital mortality	444 (21.67)	252 (21.36)	192 (22.09)	0.688
ICU admission	758 (36.99)	436 (36.95)	322 (37.05)	0.961
Adherence to SSC bundle [*n* (%)]				
Full SSC bundle	630 (30.75)	330 (27.97)	300 (34.52)	0.002*
Antibiotic administration	1356 (66.31)	751 (63.64)	605 (69.94)	0.003*
Lactate measurement	1755 (86.67)	1000 (85.91)	755 (87.69)	0.245
Blood culture drawn	1370 (67.22)	781 (66.47)	589 (68.25)	0.397
Fluid administration	1482 (72.33)	837 (70.93)	645 (74.22)	0.099
Administration of vasopressors	967 (53.63)	551 (53.03)	416 (54.45)	0.551

*SSC* Surviving Sepsis Campaign, *SOFA* Sequential Organ Failure Assessment, *APACHE* Acute Physiologic Assessment and Chronic Health Evaluation, *AIDS* acquired immunodeficiency syndrome, *GI* gastrointestinal, *ICU* intensive care unit

*p < 0.05

The volume of patients admitted to the ED during the day (n = 74,929/h, 5.2%/h) was significantly higher than the volume admitted at night (n = 50,773/h, 3.9%/h; *p* < 0.001). Supplement 3 presented result of the volume of patients per hour in each participating hospital during study period (Additional file 1: Table S3).

### Infrastructure according to duty-time and SSC bundle completion

**Fig. 2:**
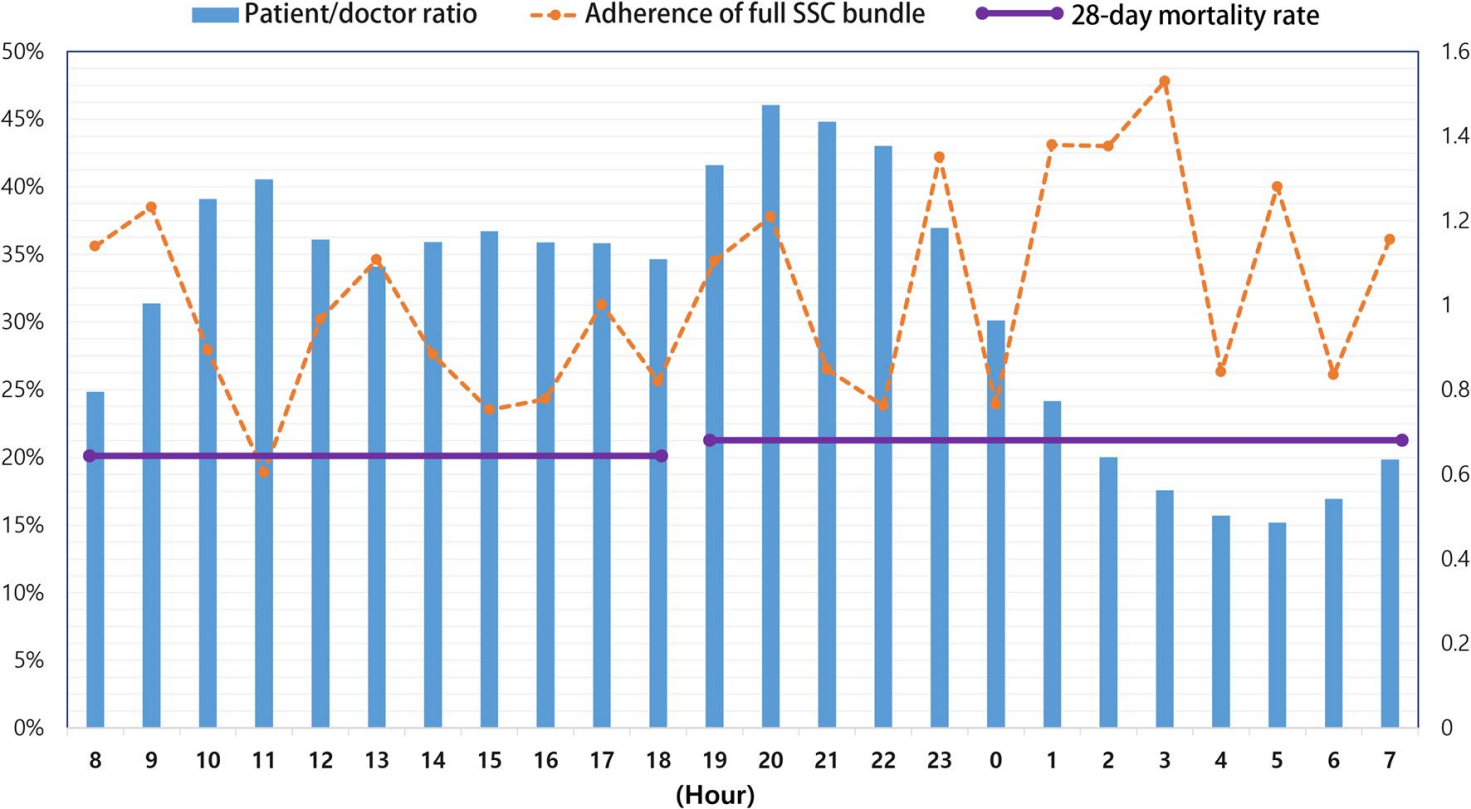
The patient to doctor ratio according to time zone in emergency department and the compliance rate for the complete Surviving Sepsis Campaign 3-h bundle. The bars indicate the patient to doctor ratio. The compliance rate for the complete sepsis bundle is shown by the dashed line, and the average 28-day mortality rate in the daytime and nighttime is represented by the solid line

Table 1 and Fig. 2 show the ratio of ED visiting patients and patients/medical staff ratio, adherence ratio of SSC bundle and mortality rate of 11 hospitals, according to the time zone (Table 1, Fig. 2). There was a significant difference in the patients/medical staff ratio daytime and nighttime, resulting in higher patient/doctor ratio and patient/nurse ratio at daytime, and lower patient/doctor ratio and patient/nurse ratio at nighttime (Table 1 and Additional file 1: Table S4). With respect to the total number of patients and the ratio of patients visiting ED, daytime was higher than nighttime. In addition, there were lower patients/medical staff ratio in group of SSC bundle completion (Additional file 1: Table S5). The number of ED beds and emergency ICU beds in each hospital are presented in Supplement 1 (Additional file 1: Table S1). All 11 participating institutions were tertiary teaching hospitals, and either regional or local emergency medical centers.

### Association between ED arrival time and compliance with the SSC 3-h bundle

Patients who arrived at the ED during the night exhibited more frequent compliance with timely antibiotic administration than those who arrived during the day, whereas the compliance rates did not differ between groups for the other components of the sepsis bundle. Based on these results, ED arrival time affected compliance with the complete SSC 3-h bundle (Table 1). The univariable logistic regression analysis showed that nighttime admission was associated with higher adherence of rapid fluid resuscitation, timely antibiotics administration and completion of SSC bundle (Additional file 1: Table S6). However, nighttime admission was not associated with timely blood sample collection for culture, vasopressor administration and lactate measurement (Additional file 1: Table S7). The multivariable logistic regression analysis revealed that, compared with patients who presented during the day, those who presented at night exhibited higher odds of compliance with the administration of antibiotics within 3 h (adjOR, 1.326; 95% CI, 1.088–1.617, *p* = 0.005) and with the complete SSC 3-h bundle (adjOR, 1.368; 95% CI, 1.115–1.678; *p* = 0.003), after adjusting for potential confounders (Tables 2, 3).

**Table 2:** The hospital stratified multivariable logistic regression analysis to identify variables significantly and independently associated with the SSC treatment bundle

Variable	Complete SSC bundle	
	AOR (95% CI)	*P*
Age (per 1 years)	1.001 (0.993–1.009)	0.823
Male (vs female)	1.0013 (0.816–1.229)	0.990
APACHE II score (per 1point)	1.0107 (0.998–1.024)	0.113
Lactate (per 1 mmol/L)	1.0122 (0.979–1.047)	0.475
C-reactive protein (mg/L)	0.999 (0.991–1.007)	0.803
Patient/doctor ratio	0.999 (0.999–1.000)	0.289
Arrival time		
Daytime	Reference	
Nighttime	1.368 (1.115–1.678)	0.003*

*SSC* Surviving Sepsis Campaign, *AOR* adjusted odds ratio, *95% CI* 95% confidence interval, *APACHE* Acute Physiologic Assessment and Chronic Health Evaluation

*P < 0.05

**Table 3:** Adjusted odds ratio from stratified multivariable logistic regression between emergency department arrival during nighttime hours and compliance with individual components of the SSC treatment bundle

Compliance with	AOR^a^ (95% CI)	*P*
	Night admission (vs day admission)	
Timely antibiotic administration	1.326 (1.088–1.617)	0.005*
Timely lactate measurement	1.145 (0.867–1.514)	0.340
Timely blood cultures	1.012 (0.787–1.302)	0.925
Timely fluid administration	1.125 (0.918–1.296)	0.147
Timely administration of vasopressors	1.137 (0.929–1.392)	0.214

*SSC* Surviving Sepsis Campaign, *OR* odds ratio, *95% CI* 95% confidence interval

^a^Adjusted for: age, sex, APACHE II score, lactate, C-reactive protein level, patients/doctor ratio

*P < 0.05

### Association between compliance with the SSC 3-h bundle and disease severity

There were significant and independent associations between the performance rate of the complete SSC 3-h bundle and nighttime admission in SOFA score < 8 and lactate < 4 with relatively lower clinical severity. However, there were no independent associations between the performance rate of the complete SSC 3-h bundle and nighttime admission in SOFA score ≥ 8 and lactate level ≥ 4 with relatively higher severity (Additional file 1: Table S8). This sensitivity analysis showed that admission time had a higher effect on SSC bundle performance in the low-severity patient group, and there was no significant difference between SSC bundle performance and ED admission time in the higher clinical severity patient group.

### Association between compliance with the SSC 3-h bundle and clinical outcomes

**Fig. 3:**
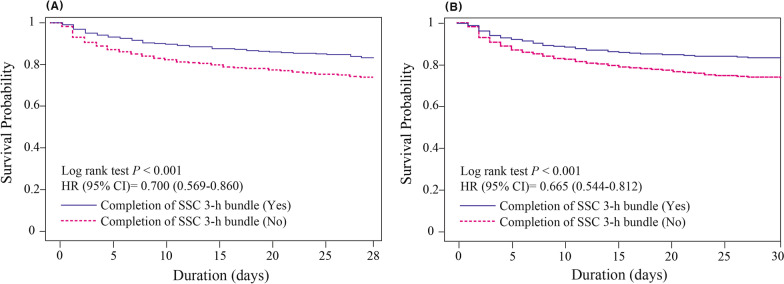
Relationships between the adherence to the SSC 3-h bundle, 28-day mortality (**A**) and in-hospital mortality (**B**). The completion of SSC 3-h bundle was significantly associated with a decreased 28-day and in-hospital mortality risk among patients with septic shock. *HR* hazard ratio, *95% CI* 95% confidence interval, *SSC* Surviving Sepsis Campaign

A total of 452 (20.1%) patients died within 28 days following ED admission and 476 (21.2%) died while hospitalized. The univariable analysis showed significant differences in the adherence of complete SSC bundle who did and did not develop 28-day and in-hospital mortality (Additional file 1: Table S9). In the multivariate Cox proportional hazards regression analyses, the hazard ratios of the intervention bundle for 28-day mortality and in-hospital mortality were 0.750 (95% CI 0.590–0.952, *p* = 0.018) and 0.714 (95% CI 0.564–0.904, *p* = 0.005), respectively, for the complete SSC 3-h bundle, and 0.742 (95% CI 0.600–0.916, *p* = 0.006) and 0.738 (95% CI 0.600–0.908, *p* = 0.004), respectively, for the timely administration of antibiotics within 3 h (Table 4). Although daytime and nighttime ED admission did not differ in terms of mortality, timely adherence to the complete 3-h bundle and to antibiotic administration was significantly associated with a decrease in 28-day and in-hospital mortality rates (Fig. 3).

**Table 4:** The hospital stratified multivariate Cox proportional-hazards regression analysis to identify variables significantly and independently associated with 28-day mortality (A) and hospital mortality rates (B)

Variable	28-day mortality			
	HR (95% CI)	*P*	HR (95% CI)	*P*
*(A)*				
Age	1.004 (0.996–1.012)	0.344	1.003 (0.995–1.011)	0.412
Male sex	1.030 (0.835–1.271)	0.781	1.037 (0.840–1.279)	0.737
APACHE II score	1.088 (1.075–1.101)	< 0.001*	1.087 (1.074–1.100)	< 0.001*
Lactate	1.105 (1.078–1.132)	< 0.001*	1.103 (1.076–1.130)	< 0.001*
C-reactive protein	1.004 (0.996–1.012)	0.357	1.004 (0.996–1.012)	0.351
Patients/doctor ratio	0.999 (0.999–1.000)	0.679	0.100 (0.999–1.000)	0.759
Source of infection				
Hepato-biliary and pancreas	0.793 (0.567–1.110)	0.177	0.785 (0.561–1.098)	0.157
Mixed source	1.146 (0.838–1.566)	0.394	1.139 (0.834–1.556)	0.413
Respiratory tract	1.288 (0.995–1.668)	0.055	1.236 (0.956–1.597)	0.106
Urinary tract	0.454 (0.309–0.667)	< 0.001*	0.453 (0.308–0.664)	< 0.001*
Timely antibiotic administration	0.742 (0.600–0.916)	0.006*		
Adherence of complete SSC bundle			0.750 (0.590–0.952)	0.018*

Variable	Hospital mortality			
	HR (95% CI)	*P*	HR (95% CI)	*P*
*(B)*				
Age	1.003 (0.996–1.011)	0.408	1.003 (0.995–1.010)	0.504
Male sex	0.978 (0.799–1.198)	0.831	0.982 (0.802–1.202)	0.860
APACHE II score	1.092 (1.079–1.106)	< 0.001*	1.091 (1.078–1.105)	< 0.001*
Lactate	1.104 (1.078–1.131)	< 0.001*	1.103 (1.077–1.130)	< 0.001*
C-reactive protein	1.005 (0.997–1.012)	0.205	1.005 (0.997–1.012)	0.220
Patients/doctor ratio	0.999 (0.999–1.000)	0.442	0.100 (0.999–1.000)	0.503
Source of infection				
Hepato-biliary and pancreas	0.750 (0.538–1.045)	0.089	0.743 (0.533–1.035)	0.079
Mixed source	1.074 (0.790–1.460)	0.648	1.073 (0.790–1.457)	0.653
Respiratory tract	1.285 (1.002–1.649)	0.049*	1.235 (0.964–1.582)	0.095
Urinary tract	0.394 (0.269–0.576)	< 0.001*	0.393 (0.268–0.574)	< 0.001*
Timely antibiotic administration	0.738 (0.600–0.908)	0.004*		
Adherence of complete SSC bundle			0.714 (0.564–0.904)	0.005*

*HR* hazard ratio, *95% CI* 95% confidence interval, *APACHE* Acute Physiologic Assessment and Chronic Health Evaluation, *SSC* Surviving Sepsis Campaign

*P < 0.05

## Discussion

The primary purpose of this study was to evaluate differences in compliance with the SSC 3-h treatment bundle according to the time of ED admission. The main finding was that patients admitted for septic shock during nighttime hours exhibited higher adherence to timely antibiotic administration and the complete SSC 3-h bundle than those admitted during daytime hours. These findings, which are based on the analysis of prospectively collected multicenter data related to the management of septic shock, contradict those of other diseases in which adverse clinical outcomes and increased mortality risk were shown to be related to nighttime or off-hour effects [8].

This study proposes the importance of the relative number of medical staff as a new key factor for improving SSC bundle performance and septic shock management. In the present study, both the total volume of patients admitted to the ED and the ratio of patients to medical staff during the day were higher than during the night. Several studies have demonstrated that the overcrowding of EDs delays sepsis management [11, 19]. For example, in a Korean study, Shin et al. reported that ED crowding significantly decreased compliance with the entire resuscitation bundle, as well as the timely implementation of the bundle elements in patients with severe sepsis [19]. Likewise, a large cohort study conducted by Peltan et al. [11] reported that each 10% increase in the ED occupancy rate was significantly associated with a 4 min delay in the door-to-antibiotic time and a 10% decrease in the probability of initiating antibiotic treatment within 3 h. Although ED overcrowding indices such as occupancy rates could not be estimated due to the retrospective nature of the present study, we were able to investigate the volume of patients visiting the ED and the number of working staff for each time period. Even though most institutions have implemented standard care protocols in the SSC, the number of patients admitted during the daytime was 35% greater than the number of nighttime admissions in the institutions participating in this study, which might explain the decreased adherence to the SSC 3-h bundle during the daytime. This was consistent with the results of a Portuguese study, which demonstrated that decreasing the number of patients led to the higher availability of medical staff, allowing for rapid antibiotic administration and vasopressor infusion [20].

In the years since the establishment of the SSC in 2002, there have been many changes in the management of sepsis, including the implementation of simplified and standardized therapeutic strategies, and comprehensive management may help reduce the marginal benefit related to the expertise of experienced clinicians and subspecialty care providers [21, 22]. Most participating institutions in the present study have applied the “Code Sepsis” protocol based on recommendations from the international guidelines and national healthcare authorities. Regardless of hospital arrival times, individual physician characteristics, and experience levels, the sepsis protocol is designed to obligate standardized management [22]. Thus, the implementation of the sepsis protocol based on the SSC might also have mitigated the “nighttime effect” in our study.

Interestingly, higher adherence of SSC bundle at nighttime was prominent in patients with relatively lower severity in our analysis. However, there was no difference in the performance rate of the complete SSC bundle between day and night admission in patients with higher severity. This is presumed because the medical staff try to maintain the strict management despite the decrease in the relative number of medical staff. Our result is in line with previous studies that medical staff tend to sustain a higher degree of monitoring in the higher severity group [23].

To date, no obvious association has been demonstrated between the period of treatment and adherence to the SSC bundle management in patients with septic shock. Regardless of the implementation of the SSC, organizational factors should be reconsidered to better understand the observed associations and to improve compliance with sepsis treatment guidelines. A retrospective study of ICU patients reported that compliance with a SSC 6-h bundle was higher at nighttime, based on the hospital arrival time; additionally, the time to address each component of the SSC 6-h bundle was also less at night than during the day [20], which is consistent with the present findings. However, that study did not provide information on the precise number of patients treated in each time period, although they suggested that a possible explanation for the findings might be the fact that fewer patients who entered the ED during nighttime hours had access to the same number of nurses as those entering during daytime [20]. Another study by Matsumura et al. reported that nighttime and weekend periods were not associated with increased in-hospital mortality in sepsis cases [10]. They demonstrated that the amount of time to administer antibiotics was significantly shorter at night than in the day, which may have contributed to reduced off-hour effects in sepsis treatment, and the number of patients with sepsis in the daytime was double that at nighttime, reducing the workload of the night staff [10].

Contrary to our results, Ranzani et al. reported that patients treated for sepsis during the daytime (based on the sepsis identification time) received more frequent lactate measurements, earlier antibiotic administration, and increased compliance with the complete SSC 3-h bundle [13]. The possible reasons for the difference between our study and the Ranzani study are as follows. First, the Ranzani study included not only ED but also general wards and ICU patients. In the wards and ICUs unlike ED, the P/D ratio and P/N ratio increase at nighttime in comparison with daytime. In general institutions, the wards and ICUs can be operated flexibly despite the decrease in the number of working staff at nighttime, and the number of hospitalized patients does not change significantly between daytime and nighttime. Therefore, general ward and ICU staffing may result in opposite effects to that of the ED regarding the P/D ratio and P/N ratio according to the day or nighttime. Second, in general, the ED maintain a relatively constant monitoring level of all patients regardless of the ED admitted time. However, the general ward may achieve a lower level of patient monitoring at nighttime in comparison with the ED [24]. In addition, several physicians stay in the ED on-site 24 h a day. In the general ward, the quality of care is more likely to decrease because the prompt accessibility of the physician is reduced at nighttime [24]. Third, the timely adherence to the complete SSC bundle may be a critical metric for EDs, affecting rankings, funding, and support of national insurance [25]. As a result, administration in the ED may be particularly sensitive to this issue in comparison to other locations of the hospital and this may positively impact upon the medical staff. This may be one of critical issues for the finding that ED admission at nighttime may result in better clinical outcomes than daytime when compared to other locations. These differences may result in inconsistencies between studies. Further study is needed to clarify the social economic effects in timely adherence to the complete SSC bundle in patients with sepsis.

As the implementation of the SSC bundle alone cannot guarantee survival in patients with sepsis, continuous effort is required by members in all institutions to mitigate the lower rates of compliance with the SSC guidelines and to improve performance. Although previous studies were conducted to investigate the difference in treatment and prognosis in patients with sepsis during daytime and nighttime, there were inconsistent results and a lack of analysis for the cause and effect of these differences. This study was intended to identify the in-depth causes from the superficial difference in SSC bundle compliance rates between daytime and nighttime. In general, there is a disadvantage of a decrease in the professionalism of medical personnel and availability of advanced medical resources, and an advantage of an increase in the relative number of medical staff (doctor/patients’ ratio and nurse/patients’ ratio) at nighttime. In the present study, there was a significant increase at nighttime for the SSC bundle compliance rate in comparison with the daytime. As this study was limited to only the sepsis management of the ED, this suggests that the increase in the relative number of medical staff such as P/D ratio and P/N ratio has a greater effect than the increase in the experienced clinicians and the availability of specialized procedures. Our study proposed a major difference from previous studies in that it suggests new key factors for improving SSC bundle performance and sepsis management.

A few studies have reported no significant association between treatment time and mortality rates [10], and the present study also did not find a significant difference in 28-day mortality rates between daytime and nighttime admissions after adjusting for confounding factors. However, an independent association was observed between SSC 3-h bundle completion and 28-day, in-hospital mortality after adjusting for clinical potential confounders, with low adherence increasing mortality risk in a manner consistent with the findings of previous studies [26]. Therefore, increasing the compliance rate of the SSC bundle during the daytime (defined as the ED arrival time) could improve the prognosis of sepsis patients, although there may be confounding pathways between SSC bundle completion and mortality that were not evaluated in the present study.

### Limitations

This study had some limitations that should be acknowledged. First, although the data were obtained from the prospective multicenter registry of consecutive patients using a standardized and predetermined protocol, the chance of missing patients exists. However, the principal investigator and the designated local research coordinator at each participating institution were responsible for verifying data accuracy and enrollment of consecutive patients, and the occurrence of missing patients was reviewed regularly. Second, the data were analyzed retrospectively. Therefore, it was difficult to completely control for potential confounding factors and to clearly determine whether the relationships between the variables were causal. Third, the enrolling criteria of the KoSS registry have been maintained without change even after the announcement of the Sepsis definition-3. However, we confirmed that there was no change in the treatment process of sepsis following the announcement of the Sepsis definition-3 in all participating institutions until December 2017. The period of this study is from November 2015 to December 2017. Fourth, we compared only the difference between day and night without comparing weekday and weekend. For weekends, there are similarities and differences to nighttime. There are similarities in the decrease in experience of medical personnel and the availability of advanced modalities. However, the difference is in the increase in the number of patients admitted to the ED on the weekend, while there are decreases in the number during the nighttime. Concerning the distortion by analyzing two off-hours with completely different trends in the number of ED admitted patients, we simply compared the characteristic of differences between nighttime and daytime in this study. Therefore, further study is needed to clarify characteristics through comparison of weekend and weekday. Finally, indicators related to medical staff's workload such as churn rate, occupancy rate, and the level of experience of individual medical staff should be also considered as very important in understanding the results of this study. Due to the retrospective nature of this study, it was impossible to obtain these data. Further study was needed to clarify effects on treatment in patients with sepsis by medical staff's workload and the level of experience of individual medical staff. Further prospective, multicenter studies are needed to identify related factors and to verify the association between ED arrival time and adherence to timely SSC bundle management in patients with septic shock.

## Conclusions

Patients experiencing septic shock who were admitted to the ED during the daytime exhibited lower SSC 3-h bundle compliance than those admitted during the nighttime. Both the higher total number of patients admitted to ED and the higher patients to medical staff ratio during the daytime may be factors that are responsible for lowering the compliance. Increasing the rate of compliance with the SSC 3-h bundle during the daytime could improve the prognosis of sepsis patients. Despite the implementation of a sepsis treatment campaign, factors that decrease bundle compliance should be reconsidered in patients experiencing septic shock.

## Supplementary Information

**Additional file 1**. Supplemental tables.

## Data Availability

The datasets used and/or analyzed during the current study are available from the corresponding author on reasonable request.

## Abbreviations

APACHE: Acute Physiologic Assessment and Chronic Health Evaluation II score
Cis: Confidential intervals
ED: Emergency department
HR: Hazard ratio
ICU: Intensive care unit
KoSS: Korean Shock Society
NEDIS: National Emergency Department Information System
ORs: Odds ratios
SOFA: Sequential Organ Failure Assessment
SSC: Surviving Sepsis Campaign
SBP: Systolic blood pressure
