# Supplementary material for: Relationship between time of emergency department admission and adherence to the Surviving Sepsis Campaign bundle in patients with septic shock
Source: Crit Care. 2022 Feb 11;26:43. doi: 10.1186/s13054-022-03899-0 (PMC8832860; doi:10.1186/s13054-022-03899-0)
Supplement: Supplementary file 1 — Additional file 1. Supplemental tables. [file 13054_2022_3899_MOESM1_ESM.docx]

**Table S1.** The infrastructures of individual hospital for the study duration.

| **Hospital** | **Infrastructure from November 2015 to December 2017** | | | | |
| --- | --- | --- | --- | --- | --- |
|  | **ED bed** | **EICU bed** | **Sepsis protocol** | **Hospital type** | **ED type** |
| **A** | 22 | 0 | Yes | Tertiary teaching hospital | Local emergency medical center |
| **B** | 18 | 0 | Yes | Tertiary teaching hospital | Local emergency medical center |
| **C** | 27 | 18 | Yes | Tertiary teaching hospital | Regional emergency medical center |
| **D** | 24 | 16 | Yes | Tertiary teaching hospital | Regional emergency medical center |
| **E** | 31 | 20 | Yes | Tertiary teaching hospital | Regional emergency medical center |
| **F** | 56 | 0 | No | Tertiary teaching hospital | Local emergency medical center |
| **G** | 26 | 17 | Yes | Tertiary teaching hospital | Regional emergency medical center |
| **H** | 33 | 0 | Yes | Tertiary teaching hospital | Local emergency medical center |
| **I** | 87 | 6 | Yes | Tertiary teaching hospital | Local emergency medical center |
| **J** | 53 | 0 | Yes | Tertiary teaching hospital | Local emergency medical center |
| **K** | 21 | 17 | No | Tertiary teaching hospital | Regional emergency medical center |


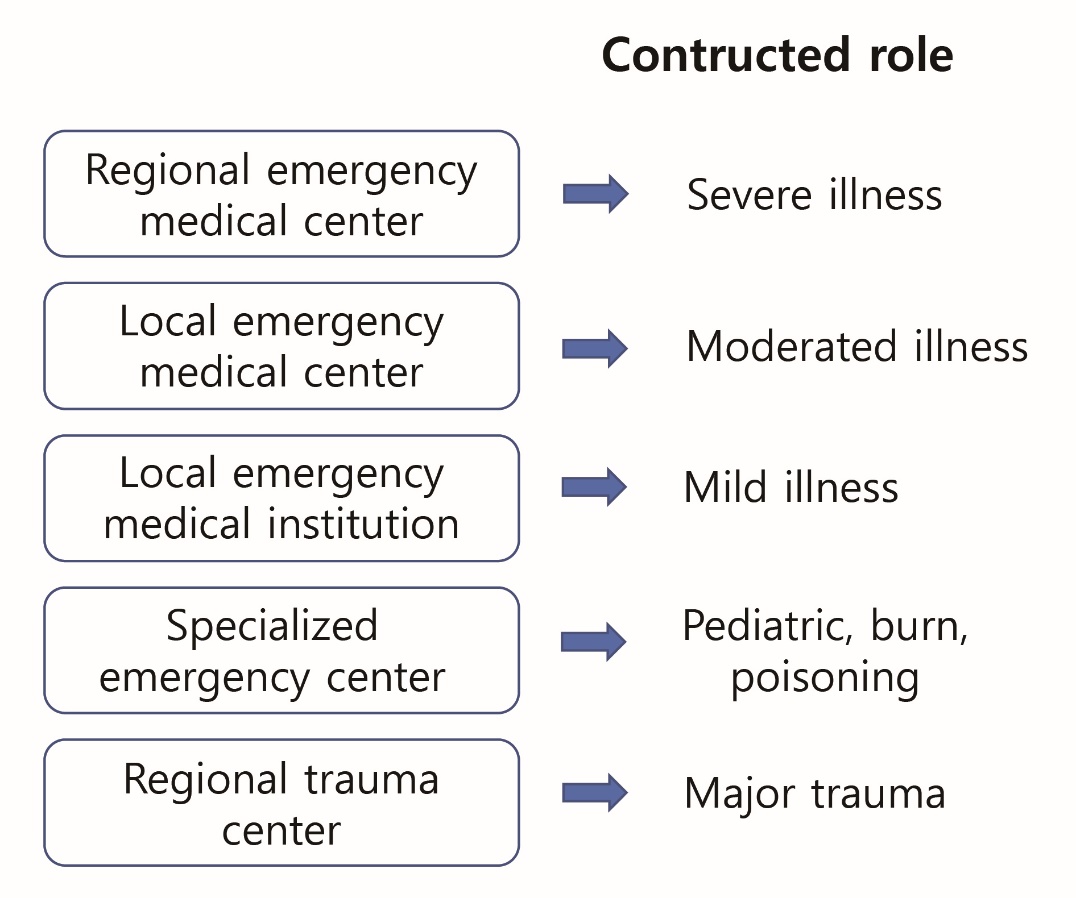


The constructed the roles according to level of emergency medical facility in South Korea.

**Table S2.** Comparison of adherence of SSC bundle according to the presence of standardized sepsis protocol.

| **Adherence of** | **Sepsis protocol** | | ***P*** |
| --- | --- | --- | --- |
|  | **Yes** | **No** |  |
| Full SSC bundle (%) | 33.0 | 29.7 | 0.136 |
| Timely antibiotic administration (%) | 69.4 | 64.9 | 0.047* |
| Timely lactate measurement (%) | 89.8 | 85.2 | 0.005* |
| Timely blood cultures (%) | 76.9 | 62.9 | <0.001* |
| Timely fluid administration (%) | 59.4 | 78.1 | <0.001* |
| Timely administration of vasopressors (%) | 42.1 | 58.1 | <0.001* |

*P<0.05

Abbreviations: SSC, Surviving Sepsis Campaign

**Table S3.** Comparison of the number of admitted patients by day and nighttime during the study period by participated institutions.

| Hospital | Number of patients from November 2015 to December 2017 | | | |
| --- | --- | --- | --- | --- |
|  | **Total** | **Daytime (per hour)** | **Nighttime (per hour)** | ***P*** |
| A | 3753±1351 | 4366±273 | 3316±1638 | 0.034* |
| B | 4517±1463 | 5123±367 | 4085±1791 | 0.053 |
| C | 5397±1981 | 6668±615 | 4489±2133 | 0.002* |
| D | 4033±1304 | 4904±360 | 3411±1384 | 0.002* |
| E | 4679±1526 | 5669±453 | 3972±1639 | 0.002* |
| F | 7550±2779 | 9501±1046 | 6156±2808 | <0.001* |
| G | 5671±2126 | 7428±799 | 4415±1866 | <0.001* |
| H | 5686±2403 | 7775±1131 | 4193±1890 | <0.001* |
| I | 9142±3341 | 11596±1197 | 7390±3289 | <0.001* |
| J | 7292±2320 | 8314±812 | 6561±2773 | 0.04* |
| K | 3119±1045 | 3585±225 | 2785±1269 | 0.037* |

*P<0.05

**Table S4.** The patient/doctor ratio (A) and the patient/nurse ratio (B) of individual hospital for the study duration.

(A)

|  | Hospital | A | B | C | D | E | F | G | H | I | J | K | overall |  |
| --- | --- | --- | --- | --- | --- | --- | --- | --- | --- | --- | --- | --- | --- | --- |
|  | Time | Average admitted patient/doctor ratio from November 2015 to December 2017 | | | | | | | | | | | | |
| Day | 09-10 h | 1.3 | 1.0 | 1.6 | 1.0 | 1.5 | 1.1 | 1.0 | 1.0 | 0.7 | 1.4 | 0.4 | 1.0 |  |
|  | 10-11 h | 1.6 | 1.2 | 2.0 | 1.2 | 1.8 | 1.5 | 1.3 | 1.1 | 1.0 | 1.8 | 0.5 | 1.3 |  |
|  | 11-12 h | 1.5 | 1.3 | 2.1 | 1.2 | 1.8 | 1.5 | 1.3 | 1.2 | 1.0 | 2.0 | 0.5 | 1.3 |  |
|  | 12-13 h | 1.3 | 1.2 | 1.8 | 1.1 | 1.5 | 1.3 | 1.2 | 1.0 | 0.9 | 1.9 | 0.5 | 1.2 |  |
|  | 13-14 h | 1.5 | 1.1 | 1.8 | 1.1 | 1.4 | 1.3 | 1.1 | 1.0 | 0.9 | 1.6 | 0.5 | 1.1 |  |
|  | 14-15 h | 1.6 | 1.1 | 1.8 | 1.1 | 1.6 | 1.3 | 1.2 | 1.0 | 0.9 | 1.7 | 0.5 | 1.1 |  |
|  | 15-16 h | 1.5 | 1.2 | 2.0 | 1.2 | 1.6 | 1.3 | 1.1 | 1.0 | 0.9 | 1.8 | 0.5 | 1.2 |  |
|  | 16-17 h | 1.6 | 1.2 | 1.9 | 1.2 | 1.5 | 1.3 | 1.0 | 0.9 | 0.9 | 1.9 | 0.5 | 1.1 |  |
|  | 17-18 h | 1.5 | 1.2 | 1.7 | 1.0 | 1.5 | 1.2 | 0.9 | 0.8 | 1.5 | 1.8 | 0.5 | 1.1 |  |
|  | 18-19 h | 1.5 | 1.2 | 1.7 | 1.0 | 1.5 | 1.1 | 0.9 | 0.7 | 1.4 | 1.6 | 0.5 | 1.1 |  |
| Night | 19-20 h | 1.8 | 1.4 | 1.9 | 1.4 | 1.7 | 1.3 | 0.9 | 1.0 | 1.5 | 2.4 | 0.6 | 1.3 |  |
|  | 20-21 h | 2.0 | 1.6 | 2.1 | 1.5 | 1.8 | 1.5 | 1.0 | 1.1 | 1.7 | 2.6 | 0.6 | 1.5 |  |
|  | 21-22 h | 2.0 | 1.5 | 2.1 | 1.5 | 1.8 | 1.4 | 1.0 | 1.1 | 1.7 | 2.6 | 0.6 | 1.4 |  |
|  | 22-23 h | 1.8 | 1.4 | 1.9 | 1.4 | 1.5 | 1.8 | 0.9 | 1.0 | 1.5 | 2.6 | 0.6 | 1.4 |  |
|  | 23-00 h | 1.5 | 1.2 | 1.7 | 1.2 | 1.4 | 1.5 | 0.8 | 0.8 | 1.3 | 2.2 | 0.5 | 1.2 |  |
|  | 00-01 h | 1.2 | 1.0 | 1.3 | 1.0 | 1.1 | 1.2 | 0.7 | 0.6 | 1.0 | 1.7 | 0.4 | 1.0 |  |
|  | 01-02 h | 1.0 | 0.8 | 1.1 | 0.8 | 0.9 | 0.9 | 0.6 | 0.5 | 0.8 | 1.4 | 0.3 | 0.8 |  |
|  | 02-03 h | 0.8 | 0.7 | 0.8 | 0.7 | 0.8 | 0.8 | 0.5 | 0.4 | 0.7 | 1.2 | 0.3 | 0.6 |  |
|  | 03-04 h | 0.7 | 0.6 | 0.7 | 0.6 | 0.7 | 0.7 | 0.4 | 0.4 | 0.6 | 1.0 | 0.2 | 0.6 |  |
|  | 04-05 h | 0.6 | 0.5 | 0.6 | 0.6 | 0.6 | 0.6 | 0.4 | 0.3 | 0.5 | 0.9 | 0.2 | 0.5 |  |
|  | 05-06 h | 0.6 | 0.5 | 0.6 | 0.5 | 0.6 | 0.6 | 0.4 | 0.3 | 0.5 | 0.9 | 0.2 | 0.5 |  |
|  | 06-07 h | 0.6 | 0.5 | 0.7 | 0.5 | 0.7 | 0.7 | 0.5 | 0.4 | 0.6 | 1.0 | 0.2 | 0.5 |  |
|  | 07-08 h | 0.6 | 0.6 | 0.7 | 0.6 | 0.8 | 0.9 | 0.6 | 0.5 | 0.7 | 1.0 | 0.2 | 0.6 |  |
|  | 08-09 h | 0.9 | 0.7 | 1.0 | 0.7 | 1.0 | 1.1 | 0.7 | 0.6 | 0.9 | 1.3 | 0.3 | 0.8 |  |

(B)

|  | Hospital | A | B | C | D | E | F | G | H | I | J | K | overall |
| --- | --- | --- | --- | --- | --- | --- | --- | --- | --- | --- | --- | --- | --- |
|  | Time | Average patient/nurse ratio from November 2015 to December 2017 | | | | | | | | | | | |
| Day | 09-10 h | 0.8 | 0.8 | 1.3 | 0.6 | 0.9 | 0.7 | 0.8 | 0.9 | 0.9 | 0.4 | 0.6 | 0.8 |
|  | 10-11 h | 0.9 | 1.1 | 1.7 | 0.7 | 1.1 | 1.0 | 1.1 | 1.2 | 1.2 | 0.5 | 0.7 | 1.0 |
|  | 11-12 h | 0.9 | 1.1 | 1.8 | 0.7 | 1.1 | 1.0 | 1.1 | 1.3 | 1.3 | 0.6 | 0.7 | 1.0 |
|  | 12-13 h | 0.8 | 1.0 | 1.5 | 0.7 | 0.9 | 0.8 | 1.0 | 1.1 | 1.2 | 0.6 | 0.7 | 0.9 |
|  | 13-14 h | 0.8 | 0.9 | 1.5 | 0.7 | 0.9 | 0.8 | 0.9 | 1.0 | 1.1 | 0.5 | 0.7 | 0.8 |
|  | 14-15 h | 0.9 | 0.9 | 1.8 | 0.7 | 1.0 | 0.8 | 1.0 | 1.1 | 1.2 | 0.5 | 0.7 | 0.9 |
|  | 15-16 h | 0.7 | 0.9 | 2.0 | 0.7 | 1.0 | 0.8 | 0.9 | 1.1 | 1.0 | 0.5 | 0.7 | 0.9 |
|  | 16-17 h | 0.8 | 0.9 | 1.9 | 0.7 | 1.0 | 0.8 | 0.9 | 1.0 | 1.0 | 0.6 | 0.7 | 0.9 |
|  | 17-18 h | 0.7 | 0.9 | 1.7 | 0.6 | 0.9 | 0.7 | 0.8 | 0.9 | 0.9 | 0.5 | 0.7 | 0.8 |
|  | 18-19 h | 0.8 | 0.9 | 1.7 | 0.6 | 0.9 | 0.7 | 0.8 | 0.8 | 0.9 | 0.5 | 0.7 | 0.8 |
| Night | 19-20 h | 0.9 | 1.1 | 1.9 | 0.7 | 1.1 | 0.8 | 0.8 | 0.9 | 1.0 | 0.6 | 0.8 | 0.9 |
|  | 20-21 h | 1.0 | 1.2 | 2.1 | 0.7 | 1.1 | 0.9 | 0.9 | 1.0 | 1.1 | 0.7 | 0.9 | 1.0 |
|  | 21-22 h | 1.0 | 1.1 | 2.1 | 0.7 | 1.1 | 0.9 | 0.9 | 1.0 | 1.0 | 0.7 | 0.9 | 0.9 |
|  | 22-23 h | 0.9 | 1.2 | 1.9 | 0.8 | 1.0 | 1.1 | 0.8 | 0.9 | 1.0 | 0.7 | 1.0 | 0.9 |
|  | 23-00 h | 1.0 | 1.0 | 1.7 | 0.7 | 0.9 | 0.9 | 0.8 | 0.7 | 1.1 | 0.6 | 0.9 | 0.9 |
|  | 00-01 h | 0.8 | 0.8 | 1.3 | 0.6 | 0.7 | 0.7 | 0.6 | 0.6 | 0.8 | 0.5 | 0.7 | 0.7 |
|  | 01-02 h | 0.6 | 0.7 | 1.1 | 0.5 | 0.6 | 0.5 | 0.5 | 0.4 | 0.7 | 0.4 | 0.6 | 0.5 |
|  | 02-03 h | 0.5 | 0.6 | 0.8 | 0.4 | 0.5 | 0.5 | 0.4 | 0.4 | 0.5 | 0.3 | 0.5 | 0.4 |
|  | 03-04 h | 0.4 | 0.5 | 0.7 | 0.3 | 0.4 | 0.4 | 0.3 | 0.3 | 0.5 | 0.3 | 0.4 | 0.4 |
|  | 04-05 h | 0.4 | 0.5 | 0.6 | 0.3 | 0.4 | 0.4 | 0.3 | 0.3 | 0.4 | 0.2 | 0.3 | 0.4 |
|  | 05-06 h | 0.4 | 0.4 | 0.6 | 0.3 | 0.4 | 0.3 | 0.3 | 0.3 | 0.4 | 0.2 | 0.3 | 0.3 |
|  | 06-07 h | 0.4 | 0.5 | 0.6 | 0.3 | 0.4 | 0.4 | 0.4 | 0.3 | 0.5 | 0.3 | 0.3 | 0.4 |
|  | 07-08 h | 0.4 | 0.5 | 0.6 | 0.3 | 0.5 | 0.4 | 0.4 | 0.4 | 0.5 | 0.3 | 0.3 | 0.4 |
|  | 08-09 h | 0.5 | 0.6 | 0.8 | 0.4 | 0.6 | 0.5 | 0.6 | 0.6 | 0.7 | 0.3 | 0.4 | 0.5 |

**Table S5.** The patient/staff ratio according to complete SSC bundle compliance.

| **Variable** | **Total** | **Adherence of full SSC bundle** | | ***P*** |
| --- | --- | --- | --- | --- |
|  |  | **Yes** | **No** |  |
| Patient/Doctor ratio | 1.19±0.44 | 1.15±0.45 | 1.20±0.43 | 0.008* |
| Patient/Nurse ratio | 0.86±0.32 | 0.82±0.31 | 0.87±0.33 | 0.003* |
| Ratio of patient per hour | 4.67±1.26 | 4.51±1.30 | 4.74±1.24 | <0.001* |

*P<0.05

Abbreviations: SSC, Surviving Sepsis Campaign

**Table S6.** The hospital stratified univariable logistic regression analysis to identify variables significantly associated with the SSC bundle.

| **Variables** | **Timely antibiotics administration** | | **Complete SSC bundle** | |
| --- | --- | --- | --- | --- |
|  | **OR (95% CI)** | ***p*** | **OR (95% CI)** | ***p*** |
| Age | 1.006(0.999-1.013) | 0.089 | 1.001(0.994-1.009) | 0.710 |
| Male sex (vs Female) | 0.999(0.825-1.209) | 0.989 | 1.013(0.828-1.240) | 0.898 |
| **Severity score** |  |  |  |  |
| SOFA score | 1.043(1.010-1.077) | 0.001* | 1.045(1.011-1.079) | 0.009* |
| APACHEⅡ score | 1.016(1.004-1.028) | 0.009* | 1.013(1.001-1.025) | 0.041* |
| **Initial vital sign** |  |  |  |  |
| SBP (per 1mmHg) | 0.995(0.991-0.999) | 0.012* | 0.980(0.975-0.985) | <0.001* |
| DBP (per 1mmHg) | 0.995(0.989-1.001) | 0.099 | 0.976(0.969-0.983) | <0.001* |
| **Past medical history** |  |  |  |  |
| Hypertension | 1.073(0.949-1.545) | 0.085 | 1.026(0.837-1.257) | 0.805 |
| Diabetes mellitus | 1.041(0.849-1.277) | 0.701 | 1.013(0.816-1.257) | 0.908 |
| Cardiovascular disease | 1.121(0.847-1.483) | 0.424 | 0.796(0.589-1.075) | 0.137 |
| Cerebraovascular disease | 1.329(0.985-1.793) | 0.063 | 1.181(0.863-1.617) | 0.299 |
| Chronic kidney disease | 0.778(0.549-1.103) | 0.159 | 0.776(0.522-1.152) | 0.208 |
| Chronic liver disease | 0.943(0.702-1.266) | 0.695 | 1.076(0.800-1.446) | 0.628 |
| Transplantation | 1.159(0.577-2.330) | 0.679 | 0.837(0.423-1.659) | 0.611 |
| Dementia | 1.145(0.768-1.708) | 0.506 | 1.167(0.754-1.806) | 0.487 |
| AIDS | 0.790(0.129-4.827) | 0.799 | 0.443(0.046-4.284) | 0.482 |
| **Source of infection** |  |  |  |  |
| GI tract | 0.843(0.643-1.107) | 0.219 | 1.018(0.763-1.357) | 0.904 |
| Hepato-biliary & pancreas | 1.050(0.814-1.354) | 0.709 | 1.138(0.884-1.466) | 0.316 |
| Respiratory | 1.443(0.854-2.336) | 0.265 | 0.896(0.705-1.137) | 0.365 |
| Soft tissue/bone/joint | 0.825(0.465-1.465) | 0.512 | 1.138(0.634-2.044) | 0.665 |
| Urinary tract | 0.841(0.663-1.067) | 0.154 | 0.998(0.768-1.296) | 0.987 |
| Mixed | 0.845(0.632-1.130) | 0.256 | 1.226(0.899-1.671) | 0.197 |
| Others | 0.863(0.567-1.313) | 0.491 | 0.922(0.598-1.421) | 0.713 |
| **Laboratory data** |  |  |  |  |
| WBC count (per 1*10^3^/μL) | 1.003(0.996-1.009) | 0.422 | 1.000(0.994-1.006) | 0.999 |
| C-reactive protein (per 1mg/L) | 1.001(0.993-1.009) | 0.768 | 0.999(0.991-1.007) | 0.749 |
| Lactate (per 1mmol/L) | 1.032(1.002-1.064) | 0.039* | 1.026(0.995-1.059) | 0.101 |
| **Arrival time** |  |  |  |  |
| Daytime | Reference |  | Reference |  |
| Nighttime | 1.345(1.109-1.630) | 0.003* | 1.381(1.131-1.686) | 0.002* |

*P<0.05

Abbreviations: SSC, Surviving Sepsis Campaign; SOFA, Sequential Organ Failure Assessment; APACHE, Acute Physiologic Assessment and Chronic Health Evaluation; SBP, systolic blood pressure; DBP, diastolic blood pressure; AIDS, acquired immunodeficiency syndrome; GI, gastrointestinal.

**Table S7.** The hospital stratified univariable logistic regression analysis to identify variables significantly associated with each component of the SSC 3h bundle

| **Variables** | **Timely lactate measurement** | | **Timely blood cultures** | | **Timely fluid administration** | | **Timely vasopressors** | |
| --- | --- | --- | --- | --- | --- | --- | --- | --- |
|  | **OR (95% CI)** | ***p*** | **OR (95% CI)** | ***p*** | **OR (95% CI)** | ***p*** | **OR (95% CI)** | ***p*** |
| Age (per 1year) | 1.005(0.996-1.015) | 0.296 | 1.001(0.992-1.010) | 0.904 | 0.993(0.985-1.001) | 0.071 | 1.004(0.997-1.011) | 0.297 |
| Male sex (vs Female) | 1.173(0.904-1.523) | 0.231 | 0.897(0.706-1.139) | 0.373 | 0.792(0.641-0.978) | 0.030* | 0.945(0.779-1.145) | 0.563 |
| **Severity score** |  |  |  |  |  |  |  |  |
| SOFA score (points) | 1.031(0.987-1.077) | 0.168 | 0.984(0.948-1.023) | 0.415 | 1.045(1.011-1.081) | 0.010* | 1.080(1.045-1.116) | <0.001* |
| APACHE score (points) | 1.024(1.007-1.042) | 0.007* | 0.985(0.971-0.999) | 0.035* | 1.009(0.996-1.022) | 0.162 | 1.004(0.992-1.016) | 0.499 |
| **Initial vital sign** |  |  |  |  |  |  |  |  |
| SBP (per 1mmHg) | 1.012(1.006-1.019) | <0.001* | 0.991(0.987-0.996) | <0.001* | 0.973(0.969-0.977) | <0.001* | 0.966(0.960-0.972) | <0.001* |
| DBP (per 1mmHg) | 1.023(1.012-1.034) | <0.001* | 0.987(0.981-0.994) | <0.001* | 0.963(0.957-0.970) | <0.001* | 0.960(0.951-0.968) | <0.001* |
| Body temperature (per 1^o^C) | 0.956(0.954-0.959) | <0.001* | 1.454(1.321-1.600) | <0.001* | 1.048(0.969-1.134) | 0.244 | 0.906(0.899-0.912) | <0.001* |
| **Past medical history** |  |  |  |  |  |  |  |  |
| Hypertension | 1.181(0.904-1.544) | 0.223 | 0.971(0.765-1.232) | 0.806 | 0.932(0.756-1.149) | 0.510 | 1.209(0.995-1.468) | 0.056 |
| Diabetes mellitus | 1.211(0.906-1.620) | 0.196 | 0.970(0.753-1.250) | 0.814 | 0.878(0.704-1.093) | 0.244 | 0.956(0.776-1.178) | 0.672 |
| Cardiovascular disease | 1.072(0.726-1.582) | 0.728 | 0.799(0.571-1.118) | 0.191 | 0.691(0.520-1.018) | 0.061 | 1.271(0.955-1.691) | 0.099 |
| Cerebraovascular disease | 1.174(0.771-1.788) | 0.454 | 0.970(0.670-1.403) | 0.870 | 1.025(0.737-1.425) | 0.885 | 1.303(0.970-1.750) | 0.079 |
| Chronic lung disease | 0.651(0.427-1.002) | 0.056 | 1.045(0.6876-1.588) | 0.837 | 0.944(0.649-1.373) | 0.763 | 1.142(0.801-1.629) | 0.463 |
| Hematologic malignancy | 0.985(0.567-1.711) | 0.957 | 1.346(0.8236-2.199) | 0.236 | 1.268(0.851-1.890) | 0.244 | 0.837(0.565-1.239) | 0.374 |
| Metastatic cancer | 0.952(0.688-1.316) | 0.766 | 1.347(0.998-1.809) | 0.057 | 1.080(0.840-1.388) | 0.549 | 0.838(0.660-1.063) | 0.145 |
| Chronic liver disease | 1.561(0.980-2.487) | 0.061 | 0.836(0.587-1.191) | 0.322 | 1.171(0.857-1.602) | 0.322 | 0.837(0.623-1.123) | 0.236 |
| Transplantation | 0.823(0.316-2.148) | 0.691 | 1.015(0.418-2.463) | 0.974 | 0.485(0.250-1.020) | 0.062 | 1.411(0.664-3.000) | 0.371 |
| Dementia | 0.804(0.487-1.328) | 0.394 | 0.994(0.605-1.632) | 0.980 | 1.291(0.795-2.095) | 0.302 | 1.186(0.792-1.775) | 0.408 |
| AIDS | 0.367(0.037-3.651) | 0.392 | 2.918(0.111-76.927) | 0.521 |  | 0.964 | 2.771(0.293-26.219) | 0.374 |
| **Source of infection** |  |  |  |  |  |  |  |  |
| GI tract | 0.959(0.657-1.340) | 0.828 | 1.047(0.7463-1.4693) | 0.790 | 1.307(0.964-1.772) | 0.085 | 0.850(0.643-1.123) | 0.253 |
| Hepato-biliary & pancreas | 1.057(0.742-1.505) | 0.760 | 1.292(0.9365-1.7830) | 0.119 | 1.061(0.808-1.391) | 0.671 | 0.874(0.679-1.125) | 0.296 |
| Soft tissue/bone/joint | 0.775(0.358-1.675) | 0.516 | 1.338(0.601-2.975) | 0.476 | 0.789(0.428-1.455) | 0.448 | 1.738(0.945-3.195) | 0.075 |
| Urinary tract | 0.949(0.682-1.320) | 0.755 | 1.004(0.739-1.364) | 0.978 | 1.350(0.985-1.800) | 0.093 | 1.116(0.874-1.425) | 0.381 |
| Mixed | 1.224(0.631-1.927) | 0.106 | 0.861(0.5943-1.248) | 0.430 | 1.164(0.840-1.613) | 0.363 | 0.908(0.727-1.153) | 0.123 |
| Others | 0.928(0.516-1.667) | 0.802 | 1.3667(0.780-2.393) | 0.275 | 0.884(0.5745-1.361) | 0.576 | 0.631(0.402-0.990) | 0.045 |
| **Laboratory data** |  |  |  |  |  |  |  |  |
| WBC count (per 1*10^3^/μL) | 1.004(0.995-1.013) | 0.398 | 0.995(0.989-1.001) | 0.092 | 1.002(0.996-1.009) | 0.495 | 1.004(0.998-1.010) | 0.202 |
| C-reactive protein (per 1mg/L) | 0.999(0.989-1.009) | 0.811 | 0.992(0.983-1.002) | 0.108 | 1.001(0.993-1.009) | 0.828 | 1.001(0.993-1.009) | 0.814 |
| Lactate (per 1mmol/L) | 1.288(1.207-1.374) | <0.001* | 0.919(0.885-0.953) | <0.001* | 0.932(0.904-0.961) | <0.001* | 0.886(0.859-0.915) | <0.001* |
| **Arrival time** |  |  |  |  |  |  |  |  |
| Daytime | Reference |  | Reference |  | Reference |  | Reference |  |
| Nighttime | 1.173(0.900-1.529) | 0.237 | 1.052(0.830-1.333) | 0.676 | 1.238(1.005-1.526) | 0.046* | 1.079(0.890-1.308) | 0.439 |

*P<0.05

Abbreviations: SSC, Surviving Sepsis Campaign; SOFA, Sequential Organ Failure Assessment; APACHE, Acute Physiologic Assessment and Chronic Health Evaluation; SBP, systolic blood pressure; DBP, diastolic blood pressure; AIDS, acquired immunodeficiency syndrome; GI, gastrointestinal.

**Table S8.** Hospital stratified multivariable logistic regression analysis to identify variables significantly and independently associated with the SSC treatment bundle according to SOFA score (A) and lactate level (B).

**(A)**

| **Variable** | **Adherence of full SSC bundle** | | | |
| --- | --- | --- | --- | --- |
|  | **SOFA < 8** | | **SOFA ≥ 8** | |
|  | **AOR (95% CI)** | ***P*** | **AOR (95% CI)** | ***P*** |
| Age (per 1 years) | 0.999(0.9903-1.0085) | 0.887 | 1.004(0.989-1.018) | 0.640 |
| Male (vs Female) | 1.082(0.8477-1.3804) | 0.528 | 0.859(0.580-1.271) | 0.446 |
| APACHEⅡ score (per 1point) | 1.014(0.9965-1.0326) | 0.115 | 1.006(0.982-1.032) | 0.619 |
| Lactate (per 1mmol/L) | 0.971(0.9277-1.0161) | 0.203 | 1.070(1.017-1.126) | 0.009* |
| C-reactive protein (per 1mg/L) | 0.998(0.9879-1.0079) | 0.675 | 1.005(0.989-1.021) | 0.554 |
| Patient/Doctor ratio | 1.000(0.9993-1.0002) | 0.300 | 1.000(0.999-1.001) | 0.581 |
| **Arrival time** |  |  |  |  |
| Daytime | Reference |  | Reference |  |
| Nighttime | 1.397(1.0970-1.7794) | 0.007* | 1.345(0.907-1.994) | 0.141 |

*P<0.05

Abbreviations: SSC, Surviving Sepsis Campaign; AOR, adjusted odds ratio; 95% CI, 95% confidence interval; APACHE, Acute Physiologic Assessment and Chronic Health Evaluation.

**(B)**

| **Variable** | **Adherence of full SSC bundle** | | | |
| --- | --- | --- | --- | --- |
|  | **Lactate < 4** | | **Lactate ≥ 4** | |
|  | **AOR (95% CI)** | ***P*** | **AOR (95% CI)** | ***P*** |
| Age (per 1 years) | 1.007(0.997-1.018) | 0.175 | 0.997(0.985-1.008) | 0.565 |
| Male (vs Female) | 0.844(0.637-1.118) | 0.237 | 1.255(0.920-1.711) | 0.151 |
| APACHEⅡ score (per 1point) | 0.993(0.973-1.014) | 0.515 | 1.023(1.005-1.041) | 0.014 |
| Lactate (per 1mmol/L) | 1.312(1.119-1.539) | <0.001* | 1.035(0.985-1.088) | 0.176 |
| C-reactive protein (mg/L) | 1.004(0.991-1.016) | 0.577 | 0.996(0.984-1.008) | 0.482 |
| Patient/Doctor ratio | 1.000(0.999-1.000) | 0.159 | 1.000(0.999-1.001) | 0.862 |
| **Arrival time** |  |  |  |  |
| Daytime | Reference |  | Reference |  |
| Nighttime | 1.451(1.092-1.928) | 0.010* | 1.281(0.947-1.733) | 0.108 |

*P<0.05

Abbreviations: SSC, Surviving Sepsis Campaign; AOR, adjusted odds ratio; 95% CI, 95% confidence interval; APACHE, Acute Physiologic Assessment and Chronic Health Evaluation.

**Table S9.** The hospital stratified univariable Cox proportional hazard regression analysis to identify variables significantly associated with the 28-day and hospital mortality.

| **Variables** | **28-day mortality** | | **Hospital mortality** | |
| --- | --- | --- | --- | --- |
|  | **HR (95% CI)** | ***p*** | **HR (95% CI)** | ***p*** |
| Age (per 1year) | 1.011(1.003-1.019) | 0.006* | 1.011(1.003-1.018) | 0.005* |
| Male sex (vs Female) | 1.358(1.111-1.661) | 0.003* | 1.312(1.080-1.593) | 0.006* |
| **Severity score** |  |  |  |  |
| SOFA score (points) | 1.188(1.153-1.224) | <0.001* | 1.187(1.153-1.223) | <0.001* |
| APACHE score (points) | 1.105(1.093-1.118) | <0.001* | 1.110(1.098-1.122) | <0.001* |
| **Initial vital sign** |  |  |  |  |
| SBP (per 1mmHg) | 1.005(1.001-1.009) | 0.018* | 1.005(1.001-1.009) | 0.022* |
| DBP (per 1mmHg) | 1.009(1.003-1.014) | 0.002* | 1.008(1.003-1.014) | 0.004* |
| Body temperature (per 1^o^C) | 0.732(0.676-0.793) | <0.001* | 0.732(0.678-0.791) | <0.001* |
| **Past medical history** |  |  |  |  |
| Hypertension | 0.942(0.774-1.146) | 0.549 | 0.949(0.784-1.148) | 0.589 |
| Diabetes mellitus | 1.148(0.962-1.525) | 0.133 | 1.158(0.935-1.528) | 0.081 |
| Cardiovascular disease | 0.975(0.735-1.294) | 0.861 | 1.014(0.773-1.331) | 0.919 |
| Cerebraovascular disease | 0.921(0.687-1.236) | 0.584 | 0.931(0.699-1.239) | 0.622 |
| Chronic kidney disease | 1.271(0.916-1.764) | 0.151 | 1.295(0.937-1.789) | 0.117 |
| Chronic liver disease | 1.247(0.983-1.791) | 0.071 | 1.382(0.977-1.947) | 0.151 |
| Transplantation | 0.251(0.063-1.011) | 0.052 | 0.366(0.117-1.142) | 0.084 |
| Dementia | 1.342(0.953-1.891) | 0.092 | 1.336(0.954-1.872) | 0.092 |
| AIDS | 0.323(0.020-5.208) | 0.426 | 0.346(0.021-5.589) | 0.455 |
| **Source of infection** |  |  |  |  |
| GI tract | 1.057(0.798-1.399) | 0.699 | 1.152(0.884-1.502) | 0.296 |
| Hepato-biliary & pancreas | 0.686(0.511-0.920) | 0.012* | 0.670(0.500-0.898) | 0.007* |
| Respiratory | 1.804(1.473-2.210) | <0.001* | 1.878(1.542-2.287) | <0.001 |
| Soft tissue/bone/joint | 0.666(0.315-1.407) | 0.287 | 0.648(0.307-1.370) | 0.256 |
| Urinary tract | 0.355(0.252-0.501) | <0.001* | 0.316(0.224-0.445) | <0.001* |
| Mixed | 1.438(1.105-1.871) | 0.007* | 1.408(1.085-1.827) | 0.01* |
| Others | 0.784(0.474-1.297) | 0.344 | 0.741(0.448-1.225) | 0.242 |
| **Laboratory data** |  |  |  |  |
| WBC count (per 1*103/μL) | 1.000(0.994-1.006) | >0.999 | 1.001(0.995-1.006) | 0.868 |
| C-reactive protein (per 1mg/L) | 1.005(0.997-1.013) | 0.201 | 1.007(0.999-1.014) | 0.063 |
| Lactate (per 1mmol/L) | 1.144(1.120-1.167) | <0.001* | 1.145(1.122-1.168) | <0.001* |
| **Adherence of SSC bundle** |  |  |  |  |
| Antibiotic administration | 0.840(0.765-0.984) | 0.041* | 0.851(0.770-0.986) | 0.044* |
| Full SSC bundle | 0.700(0.569-0.860) | <0.001* | 0.665(0.544-0.812) | <0.001* |

*P<0.05

Abbreviations: SSC, Surviving Sepsis Campaign; SOFA, Sequential Organ Failure Assessment; APACHE, Acute Physiologic Assessment and Chronic Health Evaluation; SBP, systolic blood pressure; DBP, diastolic blood pressure; AIDS, acquired immunodeficiency syndrome; GI, gastrointestinal.
